# The effects of preschool teacher’s perceived social support on autonomous motivation in China: the mediating role of emotional competence

**DOI:** 10.3389/fpsyg.2026.1796448

**Published:** 2026-07-06

**Authors:** Yanfei Cao, Mingjun Zou, Wenjing Song, Xi Lin

**Affiliations:** School of Child Development and Education, Zhejiang Normal University, Hangzhou, China

**Keywords:** autonomous motivation, emotional competence, mediating role, perceived social support, preschool teacher

## Abstract

This study examined a sample of 581 teachers from 60 diverse preschools, utilizing the Preschool Teacher Motivation Questionnaire, the Preschool Teacher Emotional Competence Questionnaire, and the Perceived Social Support Scale. The findings indicated that (a) preschool teachers’ autonomous motivation (*M* = 13.23, SD = 16.22) and perceived social support (*M* = 58.32, SD = 8.92) were above the median level, while emotional competence (*M* = 66.74, SD = 7.94) was at a medium high level, with all dimension scores exceeding the theoretical median; (b) perceived social support was significantly and positively correlated with autonomous motivation; (c) emotional competence significantly and positively predicted autonomous motivation; and (d) emotional competence fully mediated the relationship between perceived social support and autonomous motivation. These results underscore the importance of emotional competence and social support in fostering teachers’ autonomous motivation. Accordingly, this study recommends implementing strategies that target the development of emotional competence and the provision of adequate social support to effectively motivate preschool teachers.

## 1 Introduction

Teacher motivation refers to a teacher’s attitudes and values that determined their choice to choose and remain in teaching profession and shape their behavior (Han and Yin, 2016; Pratomo and Kuswati, 2022). It plays a crucial role in promoting the professional learning and development of teachers (Kumari and Kumar, 2023). Many preschool teachers reported working long hours daily and face substantial work pressure and lack of motivation (Ye and Lin, 2016). A significant proportion of these teachers have even considered leaving their profession at some point in their careers (Liang and Feng, 2004). Furthermore, teaching is considered an emotionally demanding profession, and positive relationships within the educational context not only contribute to teacher efficacy but also influence student behavior and academic performance (Lin and Wang, 2025; Olitsky, 2020; Ye et al., 2024; Yin and Lee, 2012). Preschool teachers, in particular, often have more non-teaching responsibilities and interactions with children compared to primary school teachers in many countries (OECD, 2017). This implies that preschool teachers are more likely to face emotional challenges. Moreover, the professional and emotional practices of preschool teachers are not isolated but are closely connected to their perception of and interactions with the social environment (Bronfenbrenner, 1979). Thus, it is essential that the multidimensional social environment and relationships support the professional development and emotional needs of preschool teachers to promote their motivation and passion for the job.

## 2 Literature review

### 2.1 Teacher motivation

Teacher motivation is a crucial factor that is linked to both a teacher’s professional development and their teaching performance (Carson and Chase, 2009; Kumari and Kumar, 2023; Thoonen et al., 2011). Existing research has approached teacher motivation from various perspectives, such as its definition, content, structure, motivating factors, and measurement methods (Abrami et al., 2004; Han and Yin, 2016; Osman and Warner, 2020). Through a comprehensive review of the literature, Han and Yin (2016) emphasized that teacher motivation plays a significant role in a teacher’s decision to pursue and engage in professional activities, including sustaining their teaching practices and continuously developing professionally. Notably, teacher motivation can manifest differently depending on the context, and can be categorized into two types: intrinsic motivation, which refers to the inherent drive within teachers to derive meaning from their behaviors, and extrinsic motivation, which involves behaviors driven by the expectation of external rewards or avoidance of punishment (Hsieh et al., 2022; Ryan and Deci, 2000b, 2017; Zhang et al., 2022). Extrinsic motivation can directly influence individuals’ behaviors, and can also be internalized through external pressures and emotional experiences. Organismic integration theory (OIT) outlines four forms of extrinsic motivation, namely external regulation, introjected regulation, identified regulation, and integrated regulation, which are ordered along a continuum of increasing internalization (Ryan, 1995). Externally regulated behavior is driven by rewards or punishments, whereas introjected regulation operates through inner pressures like guilt or shame. Identified regulation involves consciously valuing an activity, and at the most autonomous end, integrated regulation aligns behavior naturally with one’s core identity (Ryan and Deci, 2017). Multiple studies have reached a consensus that teacher motivation is multidimensional in nature and remains a factor that influences a teacher’s entire professional career (Han and Yin, 2016; Moè et al., 2022; Zhang et al., 2022). Previous research has highlighted that teacher motivation is influenced by both internal and external factors, including self-evaluation, effort, commitment, working conditions, professional development stages, and educational reforms (Liu et al., 2018; Mahaputra and Saputra, 2021; Ryan and Deci, 2000a,b, 2020; Sinclair, 2008). Sinclair (2008) further suggested that teachers are more likely to choose teaching out of intrinsic motivations rather than extrinsic motivations. Therefore, the levels and types of motivation exhibited by teachers can vary due to differences in social and cultural contexts as well as individual characteristics.

### 2.2 Social support

Social support is widely acknowledged in literature as a communication process through which individuals establish connections with others and their social environment (Albrecht and Adelman, 1984; Caplan, 1976). It serves as a means to satisfy fundamental psychological needs and has been found to positively impact an individual’s job satisfaction, self-efficacy, and overall wellbeing (Alon and Bergman Deitcher, 2023; Beels, 1981; Deci et al., 2006; Greenfield et al., 2023; Sarason et al., 1990). The specific content of social support is not predetermined as it depends on the ongoing situations and the needs of the recipient (Bick-har, 2019). Scholars have categorized social support into various types, including intimacy, social integration, nurturance, worth, alliance, and guidance, or personal, intra-organizational, and extra-organizational supports (Kelly et al., 1979; Sarason et al., 1983; Weiss, 1974). Perceived social support refers to an individual’s subjective perception of the overall availability of support when in need. Previous research has indicated that perceived social support is positively associated with emotions, highlighting the significance of supportive relationships in constructing and maintaining positive emotional experiences (Ahmed et al., 2010). Specifically, perceived social support has been identified as a notable predictor of subjective happiness (Gülaçtı, 2010; Sarason et al., 1990). Sarason et al. (1983) developed an instrument to assess perceived social support, comprising two main components: the perception of having adequate social supporters and the satisfaction level with the available supports. Additionally, some studies have shown that satisfaction with perceived social support outweighs the nature of the support itself (Fiorilli et al., 2017). Perceived social support, as a positive emotional experience, is linked to teachers’ working conditions and self-evaluation, which are strongly tied to motivation. Moreover, several studies have indicated that sociability plays a crucial role in career choice, as individuals with higher sociability are more likely to be influenced by intrinsic and social unity values (Pavin Ivanec and Defar, 2023).

### 2.3 Emotional competence

Emotional competence refers to the ability to effectively recognize, monitor, blunt, and control one’s own emotions in order to cope with the various challenges encountered in everyday life and professional settings (Grund and Holst, 2023; Savina et al., 2025; Tian et al., 2022). This concept is underpinned by the theory of emotional intelligence (EI) and encompasses a range of desirable behaviors such as emotional management and the building and maintenance of relationships (Boyatzis, 2016). Prior research has suggested that individuals with higher levels of EI are more likely to exhibit greater self-efficacy and experience better overall wellbeing (Chan, 2004; Coleman and Ali, 2022; Murphy and Janeke, 2009). Understanding and enhancing self-efficacy is essential for promoting motivated behavior, as emphasized by various scholars (Bandura, 1989; Deci et al., 1975; White, 1959). Given that teaching can be considered an emotional practice, the emotional competence of teachers plays a crucial role in their professional performance. A sense of control and positive emotional experiences have been found to enhance teachers’ engagement with their work and promote positive behavioral tendencies (Brunetto et al., 2012; Denzin, 2007; Hargreaves, 2000). Furthermore, it has been noted that teachers with lower emotional competence are more likely to experience dissatisfaction with perceived social support (Fiorilli et al., 2017).

## 3 The present study

Despite growing recognition of the importance of social support and emotional competence for teacher’s autonomous motivation, these three constructs have rarely been examined simultaneously in a single study. Consequently, the mediating mechanism linking social support to autonomous motivation through emotional competence remains under explored, especially among preschool teachers, who face unique emotional and relational demands. To address these gaps, the present study integrates all three constructs into a single mediation model. This integrated approach constitutes the key original contribution of this work.

The present study integrates all three constructs and is guided by two research questions (RQs):

*RQ1*: What is the current state of preschool teachers’ motivation, perceived social support, and emotional competence? This question is addressed through descriptive statistics.

*RQ2*: How are motivation, perceived social support, and emotional competence interconnected? To address RQ2, this study tests a mediation model with the following three hypotheses: (a) perceived social support has a substantial positive impact on autonomous motivation; (b) preschool teachers’ emotional competence significantly influences their autonomous motivation; and (c) emotional competence mediates the relationship between perceived social support and autonomous motivation.

## 4 Methods

### 4.1 Participants

This study employed a multimodal approach to data collection in Chinese preschool settings, utilizing both online and offline surveys. The initial pool of questionnaires consisted of 601 submissions, with 581 deemed suitable for subsequent data analysis after eliminating those deemed invalid. Prior to their participation in this investigation, preschool teachers were adequately informed about the study’s objectives, the content being examined, and the intended use of the data. To demonstrate their voluntary agreement to participate, teachers were requested to sign an informed consent form. An overview of the demographic characteristics of the study subjects is presented in Table 1.

**Table 1:** Characteristics of the participants.

Variable	*N*	%
Age		
30 years old and below	505	86.90%
31–40 years old	68	11.70%
41–50 years old	8	1.40%
Education level		
Junior college and below	98	16.90%
Bachelor	463	79.70%
Master or above	20	3.40%
Teacher experience		
0–5 years	447	76.9%
6–10 years	110	18.9%
11–15 years	17	2.9%
16–20 years	5	0.9%
More than 20 years	2	0.3%
Monthly income		
Less than RMB 2,000	44	7.6%
RMB 2,000–4,000	273	47.0%
RMB 4,001–6,000	164	28.2%
RMB 6,000 above	100	17.2%
Total	581	100%

### 4.2 Instruments

#### 4.2.1 Preschool teacher motivation questionnaire

The Preschool Teacher Motivation Questionnaire was adapted from the Autonomous Motivation for Teaching Scale (AMTS) developed by Roth et al. (2007). The AMTS was created within the framework of Self-determined Theory (SDT) and has been widely employed in researching teacher motivation due to its robust psychological foundation. Its reliability and validity have been demonstrated across diverse settings, including different countries and educational levels. To better capture the degree of individuals’ autonomy, SDT researchers categorized the four motivation types into two main dimensions: Autonomous motivation (including identified and intrinsic) and controlled motivation (including external and introjected) (Roth et al., 2007; Ryan and Connell, 1989). The AMTS assesses these four motivation types using four-item subscales, which evaluate external motivation, introjected motivation, identified motivation, and intrinsic motivation. This study adapted the AMTS to the context of early childhood education, making linguistic modifications such as changing expressions like “When I spend time on individual talks with children, I do so because I hope that parents will appreciate my work of caring for their children” and “I keep looking for interesting topics and update my teaching methods because I want the parents to be satisfied so they will not complain” (Roth et al., 2007). The questionnaire utilized a five-point Likert scale with frequency ratings ranging from “Never” to “Very often.” The internal consistency of the adapted scale was satisfactory with a Cronbach’s alpha of 0.74, and the confirmatory factor analysis (CFA) demonstrated good model fit with *χ*^2^/df = 4.67, *p* = 0.00, RMSEA = 0.08, GFI = 0.91, IFI = 0.92, and CFI = 0.92.

#### 4.2.2 Perceived social support scale

Social support scales are commonly categorized into two types: received social support, which refers to the tangible support actually received, and perceived social support, which centers on individuals’ perceptions of the received support and their satisfaction with it. The latter is believed to have a stronger association with wellbeing (Eagle et al., 2019; Graham et al., 2007; Utz, 2019). This study focuses on exploring the relationships between the target variables from the perspective of teachers and considers perceived social support as a superior predictor of an individual’s psychological state compared to received social support.

To measure preschool teacher’s perceived social support, the researchers employed the Multidimensional Scale of Perceived Social Support (MSPSS) developed by Zimet et al. (1990). This instrument has been widely utilized in various studies. The MSPSS includes three subscales, each consisting of four items, which assess perceived social support from family, friends, and significant others. Respondents rate each item on a scale ranging from 1 (strongly disagree) to 7 (strongly agree), and scores for each dimension, as well as a total individual social support score, are obtained by summing the responses.

Prior to the main study, a pilot test was conducted to examine the psychometric properties of the MSPSS. Exploratory factor analysis (EFA) revealed that one item from the Significant Others subscale exhibited substantial cross-loadings: its factor loading on the Family subscale exceeded 0.4 and was comparable to or even higher than its loading on the intended subscale. This pattern indicated a lack of discriminant validity and compromised the three-factor structure of the scale. Consequently, this item was removed from the scale. The final 11-item MSPSS consisted of the Family (4 items), Friends (4 items), and Significant Others (3 items) subscales, with all items displaying clean factor loadings (> 0.5) on their respective factors and no cross-loadings. The Cronbach’s alpha coefficient for the present scale was 0.92, and the confirmatory factor analysis (CFA) demonstrated good model fit with *χ*^2^/df = 4.70, *p* = 0.00, RMSEA = 0.08, GFI = 0.95, IFI = 0.97, and CFI = 0.97.

#### 4.2.3 Emotional competence questionnaire for preschool teachers

The development of models and measurement instruments for emotional competence is vital for advancing research in this area. Zhang conducted a systematic review of existing studies on emotional competence and utilized interviews with a large sample of proficient teachers to construct a model specifically for teachers’ emotional competence. This process led to the development of the Teacher Emotional Competence Questionnaire (Zhang, 2009). The questionnaire exhibited high reliability, validity, and cultural consistency. Building upon Zhang’s work, this study adapted the questionnaire for preschool teachers, taking into account the unique characteristics of their role. Modifications were made based on input from preschool teachers, experts in early childhood education, and official professional requirements. The adapted questionnaire consisted of 14 items covering four dimensions: emotional comprehension, emotional communication, emotional management, and emotional creativity. The scoring of the questionnaire was on a scale from 1 to 6, with each item capturing different aspects of emotional competence. The overall Cronbach alpha coefficient for the questionnaire was 0.92, suggesting high internal consistency. Confirmatory factor analysis results indicated a good model fit, as evidenced by *χ*^2^/df = 4.36, *p* = 0.00, RMSEA = 0.07, GFI = 0.93, IFI = 0.95, and CFI = 0.95.

### 4.3 Data analysis

This study utilized SPSS 25.0 and Amos 23.0 software packages to conduct data analysis. Initially, invalid samples were removed from the dataset, following which SPSS was employed to assess reliability and correlation. Subsequently, a structural equation modeling (SEM) approach was implemented using Amos. Furthermore, the mediating effect of early childhood teacher’s emotional competence on the association between perceived social support and teacher motivation was analyzed utilizing the Bootstrap test. The significance level for the tests conducted was established at *α* = 0.05.

### 4.4 Common method biases

This study utilized self-reported data, which may introduce common method biases (Hao and Lirong, 2004). To address this issue, procedural remedies such as maintaining respondent anonymity and carefully arranging the order of the questions were implemented during the pilot testing phase. Statistically, both Harman’s single-factor test and confirmatory factor analysis (CFA) were employed to assess the presence of common method bias. Harman’s single-factor test indicated a KMO value of 0.907 and a significant Bartlett’s test of sphericity *χ*^2^/df = 19.10, *p* < 0.001. The first unrotated factor accounted for only 28% of the total variance, which is well below the common threshold of 40%. The first 10 factors cumulatively explained 72% of the variance. Furthermore, a single-factor CFA model was conducted, which yielded poor model fit: *χ*^2^/df = 7.010, *p* = 0.00, RMSEA = 0.137, CFI = 0.408, AGFI = 0.347, NFI = 0.559, IFI = 0.597, TLI = 0.575. Taken together, these results suggest that common method bias does not pose a serious threat to the validity of the findings.

## 5 Results

### 5.1 Descriptive statistics

Descriptive statistics for all key study variables, including teacher motivation, perceived social support, and emotional competence, are summarized in Table 2. The mean and standard deviations for the subscales of teacher motivation and autonomous motivation were: external motivation (*M* = 12.82, SD = 3.86), introjected motivation (*M* = 12.69, SD = 3.51), identified motivation (*M* = 16.46, SD = 2.59), intrinsic motivation (*M* = 15.97, SD = 2.61), and autonomous motivation (*M* = 13.23, SD = 16.22). Compared to the theoretical median, preschool teachers reported motivation levels above the midpoint for total motivation as well as for each forms of motivation and autonomous motivation.

**Table 2:** Descriptive statistics for key study variables.

Variable	*M*	SD	Interpretation
Autonomous motivation	13.23	16.22	Above median
External	12.82	3.86	Above median
Introjected	12.69	3.51	Medium–high
Identified	16.46	2.59	Medium–high
Intrinsic	15.97	2.61	Medium–high
Perceived social support	58.32	8.92	Above median
Support from family	20.73	4.23	Above median
Support from friends	21.96	3.70	Above median
Support from significant others	15.63	2.93	Above median
Emotional competence	66.74	7.94	Medium–high
Emotional comprehension	19.13	2.82	Medium–high
Emotional communication	19.58	2.59	Medium–high
Emotional management	14.07	2.08	Medium
Emotional creativity	13.96	2.18	Medium

The paragraph presents the mean scores and standard deviations of perceived social support among preschool teachers. The results are as follows: the total score averaged at 58.32 with a standard deviation of 8.92. Furthermore, the dimensions of perceived social support were examined, including support from family (*M* = 20.73, SD = 4.23), support from friends (*M* =21.96, SD = 3.70), and support from significant others (*M* = 15.63, SD = 2.93). It is noteworthy that not only did preschool teachers perceive high levels of social support overall, but also each dimension of support exceeded the theoretical median. This implies that these teachers feel well-supported in their personal and professional relationships.

The mean scores and standard deviations of the preschool teacher’s emotional competence scores were as listed in following order: total score of emotional competence (*M* = 66.74, SD = 7.94), emotional comprehension (*M* = 19.13, SD = 2.82), emotional communication (M = 19.58, SD = 2.59), emotional management (*M* = 14.07, SD = 2.08), and emotional creativity (*M* = 13.96, SD = 2.18). The preschool teacher’s emotional competence and their dimensions scores are above the theoretical median of 3 points. They are around 5 points, indicating that preschool teacher’s emotional competence is at medium to high level.

### 5.2 Correlation analysis

The present study examined the relationships between teacher motivation, social support, and emotional competence. Correlation analysis was conducted to explore these associations, and the results are presented in Table 3. The findings demonstrate that perceived social support, as well as its individual subscales, exhibited moderate positive correlations with teacher’s autonomous motivation. Furthermore, perceived social support and its subscales were significantly and positively associated with emotional competence and its four subscales. In addition, emotional competence was significantly and positively correlated with autonomous motivation. These findings collectively suggest a robust connection between perceived social support, emotional competence, and autonomous motivation.

**Table 3:** Correlations among the study variables.

Variables	1	2	3	4	5	6	7	8	9	10
1	1									
2	0.117^**^	1								
3	0.075	0.859^***^	1							
4	0.188^***^	0.819^***^	0.521^***^	1						
5	0.011	0.768^***^	0.510^***^	0.477^***^	1					
6	0.230^***^	0.544^***^	0.438^***^	0.477^***^	0.422^***^	1				
7	0.216^***^	0.460^***^	0.356^***^	0.431^***^	0.342^***^	0.817^***^	1			
8	0.275^***^	0.437^***^	0.336^***^	0.398^***^	0.342^***^	0.850^***^	0.555^***^	1		
9	0.131^**^	0.465^***^	0.395^***^	0.374^***^	0.371^***^	0.832^***^	0.496^***^	0.722^***^	1	
10	0.105^*^	0.427^***^	0.359^***^	0.352^***^	0.335^***^	0.785^***^	0.552^***^	0.502^***^	0.579^***^	1

1 = Autonomous motivation, 2 = Perceived social support, 3 = Support from family, 4 = Support from friends, 5 = Support from significant others, 6 = Emotional competence, 7 = emotional comprehension, 8 = emotional communication, 9 = emotional management, 10 = emotional creativity, ^***^p < 0.001, ^**^p < 0.01, ^*^p < 0.05.

### 5.3 Structural equation model analysis

The initial step in the analysis involved conducting a correlation analysis to establish a basis for further examination of the mediating effect. Subsequently, structural equation models (SEM) were employed to investigate the extent to which a preschool teacher’s emotional competence mediates the relationship between perceived social support and autonomous motivation. The independent variables in this study were the teacher’s perceived social support, while autonomous motivation served as the dependent variable, and emotional competence acted as the mediator. The model, which simultaneously considered all three variables, demonstrated good fit based on statistical criteria, with *χ*^2^/df = 4.7, *p* = 0.00, RMSEA = 0.08, GFI = 0.97, AGFI = 0.93, IFI = 0.96, and TLI = 0.94.

**Figure 1:**
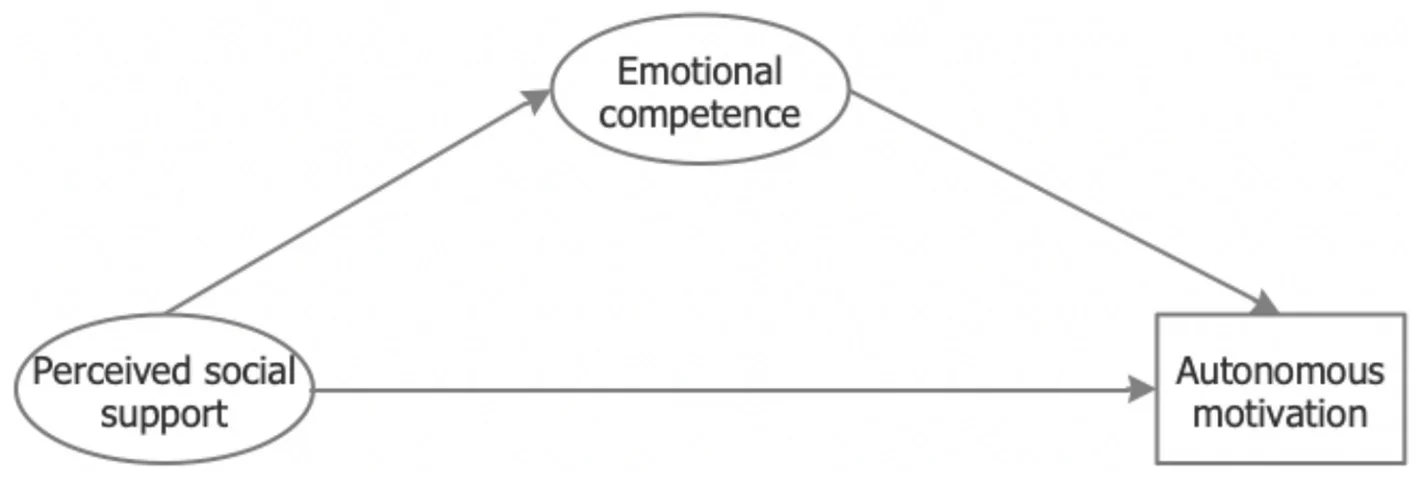
Hypothesized mediation model of perceived social support, emotional competence, and teacher motivation.

**Figure 2:**
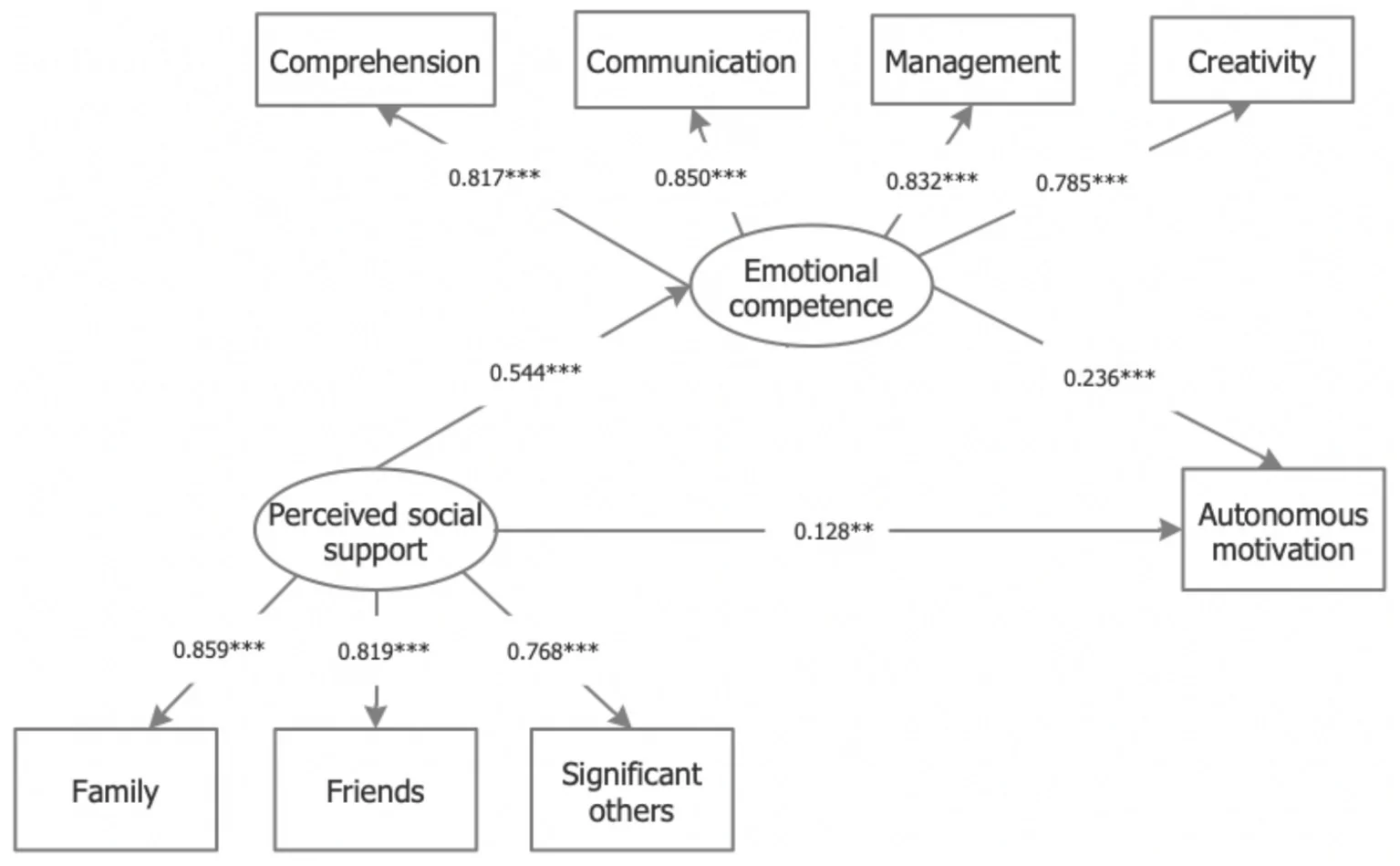
Emotional competence as a mediator of the relationships between perceived social support and teacher motivation. ^***^p < 0.001, ^**^p < 0.01.

Furthermore, using the Bootstrap method of repeated sampling 5,000 times with a 95% confidence interval for a mediating effect test, the results indicated that perceived social support was positively associated with emotional competence (*β* = 0.54, *p* < 0.001), and emotional competence was positively associated with autonomous motivation (*β* = 0.24, *p* < 0.001). The total effect of perceived social support on autonomous motivation was significant (*β* = 0.12, *p* = 0.005). However, the direct effect of perceived social support on autonomous motivation became non-significant after including the mediator (*β* = −0.01, *p* = 0.817) (see Figure 1). Critically, the indirect effect of perceived social support on autonomous motivation through emotional competence was significant, as the 95% bootstrap confidence interval did not contain zero (completely standardized indirect effect = 0.13, Boot SE = 0.02, 95% Boot CI [0.08, 0.18]). These results indicate that emotional competence fully mediated the relationship between perceived social support and autonomous motivation (Figure 2).

## 6 Discussion

This study thoroughly examined the motivation of preschool teachers by considering emotional and social support as key factors. The objective was to provide a clear understanding of the current state of motivation, perceived social support, and emotional competence among preschool teachers, as well as to explore the relationships between them. The findings indicated that preschool teachers’ motivation and autonomous motivation were moderately high. In line with the three hypotheses, this study confirmed the presence of correlations among preschool teachers’ motivation, perceived social support, and emotional competence. Furthermore, the results demonstrated that emotional competence fully mediated the relationship between perceived social support and autonomous motivation. Overall, this study enhances our understanding of the complex dynamics and interplay between motivation, social support, and emotional competence in the context of preschool teaching.

### 6.1 Current state of preschool teachers’ motivation, social support, and emotional competence

The descriptive findings revealed that preschool teachers reported moderately high levels of overall motivation, with autonomous motivation exceeding their theoretical medians. This suggests that teachers are not merely driven by external pressures; rather, they also derive genuine interest and value from their work. Regarding social support, preschool teachers reported a high level of perceived support across all three sources, family, friends, and significant others, all scoring above the theoretical median. These figures imply that teachers generally feel well-supported across both personal and professional domains. The relatively similar levels across the three sources may reflect the tight-knit nature of the interpersonal networks that preschool teachers maintain, where family members and close friends often serve overlapping supportive functions. As for emotional competence, teachers’ overall and subscale scores fell into the medium-to-high range, indicating that preschool teachers perceive themselves as fairly capable in understanding, expressing, managing, and creatively using emotions. Such a profile is encouraging, as it suggests a workforce that is generally equipped with the emotional skills necessary for the demanding interpersonal nature of early childhood education. This finding aligns with recent large-scale research on Chinese preschool teachers, which identified emotional competencies such as joy of teaching and professional value as central to teachers’ emotional networks (Xie et al., 2024) and with studies confirming moderately high emotional intelligence levels among preschool educators (Çakır and Dervişoğlu, 2025).

### 6.2 Effects of perceived social support on teacher motivation

The level of perceived social support is positively associated with the motivation of teachers. The support provided by family, friends, and significant others has a considerable impact on teachers’ motivation and autonomous motivation. In other words, when teachers feel supported in their personal and professional lives, they are more likely to be motivated and achieve better performance in their teaching. The job demands-resources model suggests that social support, as a type of job resource, negatively influences teachers’ depersonalization and intention to leave the profession. This effect is mediated by perceived relatedness. Skaalvik and Skaalvik (2018) found evidence supporting this mediation. Fiorilli et al. (2017) also confirmed that emotional exhaustion among teachers is positively associated with dissatisfaction with social support. This finding aligns with previous studies that have shown that teachers in supportive relationships with colleagues, spouses, and other significant individuals exhibit positive behaviors in their work, such as effective instruction, management, and care (Akaroğlu, 2023; Bostic et al., 2023).

The correlation between social support from friends and teacher motivation is relatively weaker compared to the other two subscales. This could be due to the fact that family members play a more prominent role in the daily lives of preschool teachers, offering tangible and consistent emotional nourishment in the form of care and affection (Utz, 2019). Furthermore, the definition of significant others can vary based on cultural and personality differences. Some researchers have found that the scales measuring friends and significant others are highly correlated (Dahlem et al., 1991; Paykani et al., 2020). In most cases, significant others are perceived as individuals who share similar experiences or are distinct from family, spouse, and friends, and can provide practical support and assistance to preschool teachers in resolving issues that may lead to demotivation (Thoits, 2011; Utz, 2019). Overall, these factors contribute to a better understanding of the relationship between social support and teacher motivation in the context of preschool education.

### 6.3 Effects of emotional competence on autonomous motivation

The present study findings provide evidence supporting the claim made by Chan (2004) that teachers’ emotional competence has a significant influence on their motivation. Specifically, the results indicate a significant correlation between all four subscales of emotional competence and autonomous motivation, including identified motivation and intrinsic motivation. This suggests that preschool teachers who excel in emotional competencies are more likely to perceive positive aspects in their work, develop a professional identity, and strive for positive and meaningful outcomes (Guay, 2022). This finding can be explained by the fact that emotional competence enhances teachers’ ability to effectively navigate the challenges of their profession and derive personal satisfaction from their role.

The efficacy of motivating teachers and supporting their professional growth remains a topic of ongoing deliberation, as evidenced by both current and previous studies. A significant aspect in this regard is the emotional competence of teachers, which pertains to the knowledge and skills required to navigate diverse situations (Mayer and Salovey, 1997). Various studies have demonstrated that training in emotional intelligence (EI) yields positive outcomes by enhancing individuals’ understanding and management of emotions. Consequently, it is imperative to incorporate EI training into both pre-service and in-service teacher education programs as a means to enhance teachers’ emotional competence (Joseph et al., 2019). Furthermore, it is essential to ensure the availability of adequate support for teachers when they encounter personal and professional challenges.

### 6.4 The mediation role of emotional competence

Confirming the third hypothesis, emotional competence fully mediated the relationship between perceived social support and autonomous motivation. This suggests that perceived social support influences teachers’ autonomous motivation entirely through emotional competence. Emotional competence is considered a crucial component of social support, with emotional support being a common manifestation of social assistance that involves qualities such as care, empathy, and a sense of belonging (Thoits, 1985). Recent research with Chinese kindergarten teachers further confirms that emotional support competence is strongly linked to teachers’ prosocial motivation and professional mission (Lin et al., 2025). It is important to note that the effectiveness of the received support is contingent upon the specific demands and appreciation of the recipient: the positive impact of social support is only observable when the support resources are aligned with the individual’s adaptive requirements (Wilcox and Vernberg, 1985). This principle resonates with recent evidence from a systematic review indicating that the long-term benefits of social-emotional competence interventions are contingent upon contextual factors such as supportive school environments, sustained leadership, and adequate resource allocation (Ved and Kareem, 2026). Together, these findings reinforce the notion that social support is not simply an external resource passively received by teachers, but a dynamic resource whose effectiveness depends on both the recipient’s emotional capabilities and the alignment between support and individual needs (Cervellione et al., 2025).

## 7 Conclusion

The present study aimed to investigate the interrelationships among preschool teachers’ autonomous motivation, perceived social support, and emotional competence. The study confirmed the three initial hypotheses put forth. The findings indicate that preschool teacher’s autonomous motivation is associated with both perceived social support and individual emotional competence. Moreover, both perceived social support and emotional competence positively predict autonomous motivation, with emotional competence serving as a full mediator in the relationship between the other two variables. These results suggest that teachers’ motivation cannot be solely attributed to their work concepts, abilities, and attitudes, but is also influenced by the quantity and intensity of social support they receive and perceive, as well as their emotional intelligence. Consequently, in order to enhance teachers’ autonomous motivation, it is crucial to consider providing them with adequate support and to simultaneously strengthen the development of their emotional abilities through pre-service and post-service training programs.

This study has verified the role of emotional competence as a mediator in the connection between perceived social support and autonomous motivation. However, the study did not investigate whether this mediation occurs through a direct impact on the fundamental psychological needs or by altering cognitive pathways. The former hypothesis suggests that emotional competence directly influences teachers’ psychological needs for autonomy and relatedness, while the latter hypothesis proposes that emotional competence further influences individuals’ evaluative processes and causal orientations. Therefore, the specific mediating mechanisms will be examined in future research. Overall, this study offers valuable insights into the relationship between emotional competence, perceived social support, and teacher motivation, but additional investigations are needed to fully understand the underlying processes.

Moreover, the findings of this study indicate a significant correlation between teacher motivation and demographic variables such as marital status, age, and years of teaching experience. However, it remains unclear whether these demographic factors directly or indirectly impact autonomous motivation, as this aspect has not been thoroughly examined. Additionally, it is crucial to recognize that the autonomous motivation, social support, and emotional competence of preschool teachers are not fixed traits, but rather temporary states within their professional development. Indeed, a teacher’s motivation is subject to fluctuations influenced by factors such as self-identity, teacher education, efficacy, and the external environment. Previous research (Bas, 2022; Richardson, 2003; Tschannen-Moran and Hoy, 2001) supports the notion that practical and effective measures can be implemented to enhance the motivation of preschool teachers. However, it is important to note that this study did not implement any interventions to modify teachers’ motivation status. Consequently, the current study’s conclusions should be regarded as a starting point, necessitating further research and investigation in this area.

## Data Availability

The raw data supporting the conclusions of this article will be made available by the authors, without undue reservation.
