# Correction: The effects of preschool teacher's perceived social support on autonomous motivation in China: the mediating role of emotional competence

**DOI:** 10.3389/fpsyg.2026.1954217

**Published:** 2026-08-25

**Authors:** Yanfei Cao, Mingjun Zou, Wenjing Song, Xi Lin

**Affiliations:** School of Child Development and Education, Zhejiang Normal University, Hangzhou, China

**Keywords:** autonomous motivation, emotional competence, mediating role, perceived social support, preschool teacher

In the published article, in **Abstract** section, the means (M) and standard deviations (SDs) reported for perceived social support and emotional competence were incorrect because values from an earlier version had inadvertently been retained.

This has been corrected to read:

“The findings indicated that (a) preschool teachers' autonomous motivation (M = 13.23, SD = 16.22) and perceived social support (M = 58.32, SD = 8.92) were above the median level, while emotional competence (M = 66.74, SD = 7.94) was at a medium-high level, with all dimension scores exceeding the theoretical median; (b) perceived social support was significantly and positively correlated with autonomous motivation; (c) emotional competence significantly and positively predicted autonomous motivation; and (d) emotional competence fully mediated the relationship between perceived social support and autonomous motivation.”

In addition, there was a mistake in Table 1 “Characteristics of the participants,” as published. The percentage of teachers with more than 20 years of teaching experience was incorrectly reported as 2.3%; the correct value is 0.3%. The corrected Table 1 appears below.

**Table 1:** Characteristics of the participants.

Variable	*N*	%
Age		
30 years old and below	505	86.90%
31–40 years old	68	11.70%
41–50 years old	8	1.40%
Education level		
Junior college and below	98	16.90%
Bachelor	463	79.70%
Master or above	20	3.40%
Teacher experience		
0–5 years	447	76.9%
6–10 years	110	18.9%
11–15 years	17	2.9%
16–20 years	5	0.9%
More than 20 years	2	0.3%
Monthly income		
Less than RMB 2,000	44	7.6%
RMB 2,000–4,000	273	47.0%
RMB 4,001–6,000	164	28.2%
RMB 6,000 above	100	17.2%
Total	581	100%

There was also a mistake in Table 2, “Descriptive statistics for key study variables,” as published. Due to a typographical error, the interpretation of “Support from significant others” was incorrectly reported as “Below median.” The correct interpretation is “Above median.” The corrected Table 2 appears below.

**Table 2:** Descriptive statistics for key study variables.

Variable	*M*	*SD*	Interpretation
Autonomous motivation	13.23	16.22	Above median
External	12.82	3.86	Above median
Introjected	12.69	3.51	Medium–high
Identified	16.46	2.59	Medium–high
Intrinsic	15.97	2.61	Medium–high
Perceived social support	58.32	8.92	Above median
Support from family	20.73	4.23	Above median
Support from friends	21.96	3.70	Above median
Support from significant others	15.63	2.93	Above median
Emotional competence	66.74	7.94	Medium–high
Emotional comprehension	19.13	2.82	Medium–high
Emotional communication	19.58	2.59	Medium–high
Emotional management	14.07	2.08	Medium
Emotional creativity	13.96	2.18	Medium

Further, the standard deviation for identified motivation in Section 5.1 was incorrectly reported as 2.58 due to a typographical error. The correct value is 2.59, as presented in Table 2. The original data and statistical analyses were correct, and this error does not affect the results or conclusions of the article.

A correction has been made to the section 5, “**Results**,” subsection 5.1 “**Descriptive statistics**,”

Paragraph 1:

“Descriptive statistics for all key study variables, including teacher motivation, perceived social support, and emotional competence, are summarized in Table 2. The mean and standard deviations for the subscales of teacher motivation and autonomous motivation were: external motivation (M = 12.82, SD = 3.86), introjected motivation (M = 12.69, SD = 3.51), identified motivation (M = 16.46, SD = 2.59), intrinsic motivation (M = 15.97, SD = 2.61), and autonomous motivation (M = 13.23, SD = 16.22). Compared to the theoretical median, preschool teachers reported motivation levels above the midpoint for total motivation as well as for each forms of motivation and autonomous motivation.”

The means and standard deviations for the total perceived social support score and its three dimensions in Section 5.1, Paragraph 2, were incorrectly reported because values from an earlier version of the statistical results were inadvertently retained in the text. The corresponding values presented in Table 2 were correct. This error does not affect the statistical analyses, results, or conclusions of the article.

A correction has been made to the section 5, “**Results**,” subsection 5.1 “**Descriptive statistics**,”

Paragraph 2:

“The paragraph presents the mean scores and standard deviations of perceived social support among preschool teachers. The results are as follows: the total score averaged at 58.32 with a standard deviation of 8.92. Furthermore, the dimensions of perceived social support were examined, including support from family (M = 20.73, SD = 4.23), support from friends (M = 21.96, SD = 3.70), and support from significant others (M = 15.63, SD = 2.93). It is noteworthy that not only did preschool teachers perceive high levels of social support overall, but also each dimension of support exceeded the theoretical median. This implies that these teachers feel well-supported in their personal and professional relationships.”

The means and standard deviations for overall emotional competence and its four dimensions were incorrectly reported in Section 5.1, Paragraph 3, because values from an earlier version of the statistical results were inadvertently retained in the text. The corresponding values presented in Table 2 were correct. This error does not affect the statistical analyses, results, or conclusions of the article.

A correction has been made to the section 5, “**Results**,” subsection 5.1 “**Descriptive statistics**,”

Paragraph 3:

“The mean scores and standard deviations of the preschool teacher's emotional competence scores were as listed in following order: total score of emotional competence (M = 66.74, SD = 7.94), emotional comprehension (M = 19.13, SD = 2.82), emotional communication (M = 19.58, SD = 2.59), emotional management (M = 14.07, SD = 2.08), and emotional creativity (M = 13.96, SD = 2.18). The preschool teacher's emotional competence and their dimensions scores are above the theoretical median of 3 points. They are around 5 points, indicating that preschool teacher's emotional competence is at medium to high level.”

The mediating role of emotional competence was incorrectly described as partial because the interpretation from an earlier version of the mediation analysis was inadvertently retained in the text. The final mediation analysis indicated that emotional competence fully mediated the relationship between perceived social support and autonomous motivation. Accordingly, “partially” has been corrected to “fully.” The original data were unchanged, and the corrected statement is consistent with the final statistical analysis.

A correction has been made to the section 6, “**Discussion**,” subsection 6.4, “**The mediation role of emotional competence**,”

Paragraph 1:

“Confirming the third hypothesis, emotional competence fully mediated the relationship between perceived social support and autonomous motivation. This suggests that perceived social support influences teachers' autonomous motivation entirely through emotional competence. Emotional competence is considered a crucial component of social support, with emotional support being a common manifestation of social assistance that involves qualities such as care, empathy, and a sense of belonging (Thoits, 1985). Recent research with Chinese kindergarten teachers further confirms that emotional support competence is strongly linked to teachers' prosocial motivation and professional mission (Lin et al., 2025). It is important to note that the effectiveness of the received support is contingent upon the specific demands and appreciation of the recipient: the positive impact of social support is only observable when the support resources are aligned with the individual's adaptive requirements (Wilcox and Vernberg, 1985). This principle resonates with recent evidence from a systematic review indicating that the long-term benefits of social-emotional competence interventions are contingent upon contextual factors such as supportive school environments, sustained leadership, and adequate resource allocation (Ved and Kareem, 2026). Together, these findings reinforce the notion that social support is not simply an external resource passively received by teachers, but a dynamic resource whose effectiveness depends on both the recipient's emotional capabilities and the alignment between support and individual needs (Cervellione et al., 2025).”

Emotional competence was incorrectly described as a partial mediator in the Conclusion because the interpretation from an earlier version of the mediation analysis was inadvertently retained in the text. The final mediation analysis indicated that emotional competence was a full mediator in the relationship between perceived social support and autonomous motivation. Accordingly, “partial mediator” has been corrected to “full mediator.” The original data were unchanged, and the corrected statement is consistent with the final statistical analysis.

A correction has been made to the section 7, “**Conclusion**,”

Paragraph 1:

“The present study aimed to investigate the interrelationships among preschool teachers' autonomous motivation, perceived social support, and emotional competence. The study confirmed the three initial hypotheses put forth. The findings indicate that preschool teacher's autonomous motivation is associated with both perceived social support and individual emotional competence. Moreover, both perceived social support and emotional competence positively predict autonomous motivation, with emotional competence serving as a full mediator in the relationship between the other two variables. These results suggest that teachers' motivation cannot be solely attributed to their work concepts, abilities, and attitudes, but is also influenced by the quantity and intensity of social support they receive and perceive, as well as their emotional intelligence. Consequently, in order to enhance teachers' autonomous motivation, it is crucial to consider providing them with adequate support and to simultaneously strengthen the development of their emotional abilities through pre-service and post-service training programs. ”

The original version of this article has been updated.

